# Association between hospital palliative care team intervention volume and patient outcomes

**DOI:** 10.1007/s10147-024-02574-4

**Published:** 2024-06-24

**Authors:** Hiroaki Abe, Masahiko Sumitani, Hiroki Matsui, Reo Inoue, Kiyohide Fushimi, Kanji Uchida, Hideo Yasunaga

**Affiliations:** 1 Department of Pain and Palliative Medicine, The University of Tokyo Hospital, 7-3-1 Hongo, Bunkyo-ku, Tokyo, 113-8655 Japan; 2 Department of Clinical Epidemiology and Health Economics, School of Public Health, The University of Tokyo, Tokyo, Japan; 3 Department of Anesthesiology and Pain Relief Center, The University of Tokyo Hospital, Tokyo, Japan; 4 Department of Health Policy and Informatics, Tokyo Medical and Dental University, Tokyo, Japan

**Keywords:** Palliative care team, Volume-outcome association, Cancer patients, Delirium

## Abstract

**Background:**

The benefits of palliative care in patients with advanced cancer are well established. However, the effect of the skills of the palliative care team (PCT) on patient outcomes remains unclear. Our aim was to evaluate the association between hospital PCT intervention volume and patient outcomes in patients with cancer.

**Methods:**

A retrospective cohort study was conducted using a nationwide inpatient database in Japan. Patients with cancer receiving chemotherapy and PCT intervention from 2015 to 2020 were included. The outcomes were incidence of hyperactive delirium within 30 days of admission, mortality within 30 days of admission, and decline in activities of daily living (ADL) at discharge. The exposure of interest was hospital PCT intervention volume (annual number of new PCT interventions in a hospital), which was categorized into low-, intermediate-, and high-volume groups according to tertiles. Multivariate logistic regression and restricted cubic-spline regression were conducted.

**Results:**

Of 29,076 patients, 1495 (5.1%), 562 (1.9%), and 3026 (10.4%) developed delirium, mortality, and decline in ADL, respectively. Compared with the low hospital PCT intervention volume group (1–103 cases/year, n = 9712), the intermediate (104–195, n = 9664) and high (196–679, n = 9700) volume groups showed significant association with lower odds ratios of 30-day delirium (odds ratio, 0.79 [95% confidence interval, 0.69–0.91] and 0.80 [0.69–0.93], respectively), 30-day mortality (0.73 [0.60–0.90] and 0.59 [0.46–0.75], respectively), and decline in ADL (0.77 [0.70–0.84] and 0.52 [0.47–0.58], respectively).

**Conclusion:**

Hospital PCT intervention volume is inversely associated with the odds ratios of delirium, mortality, and decline in ADL among hospitalized patients with cancer.

**Supplementary Information:**

The online version contains supplementary material available at 10.1007/s10147-024-02574-4.

## Introduction

The incidence of cancer, one of the leading causes of death worldwide, is steadily increasing in many countries owing to the aging of the population and advancements in cancer screening and treatment [1]. According to prevalence estimates in 2022, the estimated number of people who were alive within 5 years following a cancer diagnosis was 53.5 million [2]. Patients with advanced cancer often experience physical and psychological distress, which can significantly affect their quality of life (QOL), and palliative care is required to improve their QOL [3, 4].

Palliative care focuses on improving the QOL of patients with life-limiting illnesses and their families. Palliative care is provided by a multidisciplinary team of healthcare professionals including doctors, nurses, pharmacists, psychologists, and social workers. Palliative care teams (PCTs) work together to relieve physical, psychological, and spiritual symptoms, as well as other concerns experienced by patients and their families [3, 4]. The benefits of palliative care in patients with advanced cancer are well established. A previous randomized controlled trial revealed that providing early palliative care to patients with advanced lung cancer can improve the QOL and depressive symptoms compared with standard care [5]. Although fewer patients in the early palliative care group received aggressive end-of-life care, including chemotherapy, compared with those in the standard care group, the median survival was longer for patients who received early palliative care. A previous randomized controlled trial assessed the effect of integrated palliative care on outcomes in patients with acute myeloid leukemia receiving intensive chemotherapy and revealed that integrated palliative care led to significant improvements in QOL and psychological distress [6]. Another randomized controlled trial investigating the impact of in-home palliative care intervention in terminally ill patients revealed that patients receiving in-home palliative care reported greater improvement in satisfaction with care and were less likely to visit the emergency department or be admitted to the hospital than those receiving usual care, resulting in significantly lower costs of care [7]. A randomized controlled trial that evaluated the effect of early integrated palliative care on QOL and the use of health care resources near the end of life in patients with advanced cancer reported that the QOL of patients receiving integrated palliative care was significantly higher compared with that of those receiving usual care. [8]

Although the importance of palliative care in improving patient outcomes in patients with advanced cancer has been established, it remains unclear whether PCT skills affect patient outcomes. Inappropriate prescription of opioids can lead to respiratory depression and death in patients with cancer [9]. Overuse of antipsychotics can also lead to oversedation and aspiration pneumonia in patients with cancer [10, 11]. Delayed treatment of delirium may increase the incidence of pneumonia and mortality [12]. Thus, it can be assumed that PCT skills can affect the patient outcome. However, evaluating the association between PCT skills and patient outcomes remains difficult owing to the difficulty in assessing PCT skills. Previous studies investigating the impact of surgical skill on patient outcomes often evaluated the association between annual hospital procedural volume and patient outcomes [13]. Thus, the association between PCT skills and patient outcome could be assessed using hospital PCT intervention volume as an alternative to PCT skills.

We hypothesized that higher PCT skills lead to better patient outcomes. Hence, this study investigated the association between hospital PCT intervention volume and the outcomes of hospitalized patients with cancer receiving chemotherapy using nationwide administrative data in Japan.

## Materials and methods

### Data source

This study was approved by the Institutional Review Board and Ethics Committee of The University of Tokyo (Institutional Review Board number: 3501). The requirement for obtaining written informed consent was waived as the study was a secondary analysis of anonymous administrative data.

Patient data were extracted from the Japanese Diagnosis Procedure Combination database, a national database of administrative claims and discharge abstracts in Japan. Eighty-two university hospitals in Japan participate mandatorily in the database, whereas over 1600 community hospitals participate voluntarily. The database included the administrative data of 11 million inpatients in 2020, accounting for approximately 80% of all acute care inpatients in Japan [14]. The details of the database have been described previously. [15]

The database includes the following data: hospital information (hospital identifier, hospital type, and number of hospital beds), patient information (patient identifier, age, sex, height, body weight, and smoking status), information at admission (purpose of hospitalization, primary diagnosis, comorbidities, route of hospitalization, activities of daily living [ADL]), information regarding treatments (surgery, anesthesia, medication, and blood transfusion), information at discharge (discharge status and ADL), and costs. Diagnoses are recorded using the International Classification of Diseases, 10th Revision (ICD-10) codes and Japanese text. All medical procedures are encoded using the original Japanese medical procedure codes.

A previous validation study on the Japanese Diagnosis Procedure Combination database revealed that the recorded procedures and drugs had high sensitivity and specificity, whereas the recorded diagnoses of common diseases, including malignant tumors, cardiac diseases, renal diseases, and stroke, had moderate sensitivity and high specificity [15].

### Population

This study included hospitalized patients with cancer aged ≥ 20 years who received chemotherapy and PCT intervention within 2 days of admission between January 1, 2015, and December 31, 2020. The present study only included hospitalized patients with cancer receiving chemotherapy to obtain a relatively homogeneous population. PCT intervention was identified using the registry of the Japanese Medical Procedure Code A226-2. Patients with cancer who were concurrently diagnosed with schizophrenia (ICD-10 codes F20.x–F29.x) were excluded from this study as it was difficult to distinguish whether these patients were receiving antipsychotics for schizophrenia or delirium. Emergency hospitalized patients were also excluded because receiving chemotherapy within 2 days of emergency hospitalization was considered an exceptional case.

### Exposure of interest

The exposure of interest was the hospital PCT intervention volume, which was defined as the annual number of new PCT interventions performed in a hospital. The PCT intervention volume was calculated using the hospital identifier, and the hospitals were categorized into low-, intermediate-, or high-volume groups according to their tertiles.

### Outcomes

The primary outcome was the incidence of hyperactive delirium within 30 days of admission. Hyperactive delirium was identified by the administration of haloperidol or risperidone ≥ 2 days after admission. This delirium identification algorithm was adopted in this study as a similar identification algorithm achieved sufficient validity for use in claims-based databases [16]. The secondary outcomes were mortality within 30 days of admission and a decline in ADL at discharge. Decline in ADL was identified by comparing the Barthel Index at admission and discharge. Death was categorized as a decline in ADL.

### Potential confounders

The potential confounders used for the regression analyses included the demographic and hospital characteristics of the patients, comorbidities, and medications prescribed at admission. These variables were selected from the pretreatment factors that could be associated with the incidence of delirium, mortality, and decline in ADL, according to clinical judgment and the existing literature [17–26]. Demographic characteristics included the year of admission, sex, age, body mass index, smoking status, and Barthel Index of the patients. The Barthel Index is a measure of the functional independence of individuals in ADL [27]. The total score on the Barthel Index ranges from 0 to 100. Higher scores on the Barthel Index indicate greater functional independence, with a score of 100 indicating that the individual was completely independent in all ADLs. The hospital characteristics included the type of hospital (academic/community), number of hospital beds, and chemotherapy volume (i.e., the annual number of inpatient chemotherapies in a hospital). The hospitals were categorized into two groups based on the median number of hospital beds, whereas the chemotherapy volume was categorized into three groups according to tertiles. Comorbidities included the Charlson Comorbidity Index, dementia (ICD-10 codes F00.x–F03.x or use of anti-dementia agents), brain metastasis (C79.3), and type of cancer (lung cancer, C33.x-C39.x; lower gastrointestinal cancer, C17.x-C21.x; upper gastrointestinal cancer, C15.x-C16.x; leukemia and lymphoma, C81.x-C96.x; and other cancers). The Charlson Comorbidity Index, a score used to classify the comorbid conditions of a patient, was calculated using Quan’s algorithm [28, 29]. Medications included antibiotics, opioids, gabapentinoids, and hypnotics. Patients who received these medications within 2 days of admission were considered to be on medication.

### Statistical analysis

Multivariate logistic regression analyses were performed to estimate the odds ratios for 30-day delirium, 30-day mortality, and decline in ADL using the hospital PCT intervention volume and potential confounders as independent variables. The odds ratios were calculated for the intermediate and high hospital PCT intervention volume groups using the low hospital PCT intervention volume group as the reference. Multicollinearity was assessed using the variance inflation factor, with a variance inflation factor larger than 10 indicating deleterious multicollinearity [30].

Restricted cubic spline regression analyses were conducted subsequently to evaluate the potential non-linear associations between continuous hospital PCT intervention volume and outcomes. Restricted cubic spline regression analysis is advantageous over standard categorical regression analysis in that it circumvents the power loss associated with categorization [31, 32]. Three hospital PCT intervention volume points (the 10th, 50th, and 90th percentiles) were used as knots in the restricted cubic spline regression analysis. The odds ratios for each value of hospital PCT intervention volume were calculated using the lowest hospital PCT intervention volume (1 case/year) as the reference value. The hospital PCT intervention volume and the same potential confounders used in the logistic regression described above were used as independent variables in the restricted cubic spline regression analyses.

Categorical variables are presented as numbers (percentages) and were compared using the Chi-square test. Odds ratios are presented as 95% confidence intervals (CI). All reported *P* values were two-sided, and *P* values of < 0.05 were considered statistically significant. All statistical analyses were performed using Stata/SE 17.0 (Stata Corp., College Station, Texas, USA). This manuscript adhered to the Strengthening the Reporting of Observational Studies in Epidemiology guidelines.

## Results

**Fig. 1:**
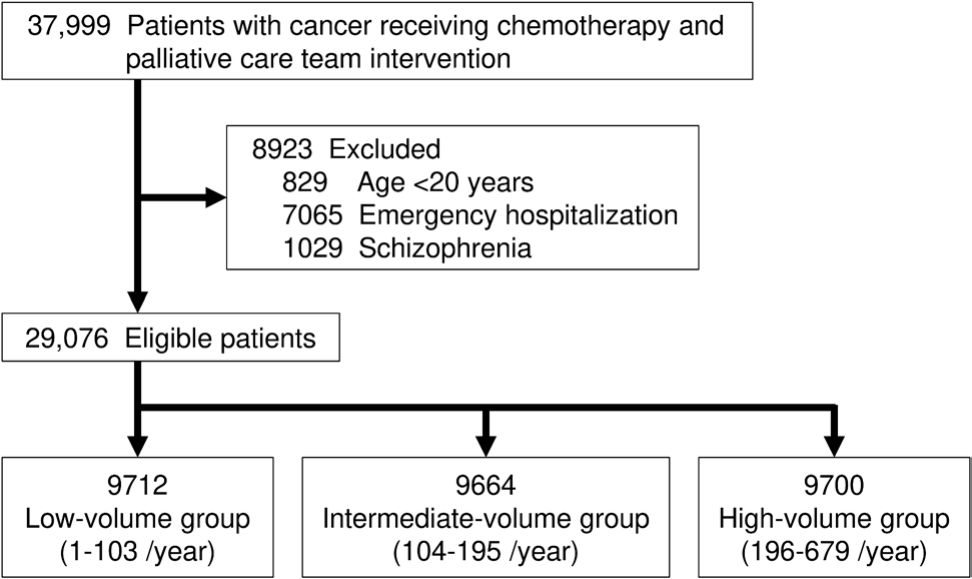
Flow chart of patient inclusion and exclusion in the study sample

In total, 37,999 hospitalized patients with cancer who received chemotherapy and initiated PCT intervention within 2 days of admission between January 1, 2015, and December 31, 2020, were identified from the database. After applying the exclusion criteria (Fig. 1), 29,076 patients were included in this study [13,479 (46.4%) male; mean age, 61.4 years (standard deviation, 13.4); median length of hospital stay, 12 days (interquartile range, 5–24)]. The incidence of 30-day delirium, 30-day mortality, and decline in ADL were 5.1% (n = 1495), 1.9% (n = 562), and 10.4% (n = 3026), respectively. The hospital PCT intervention volumes were calculated for each hospital, and hospitals were categorized into three volume groups based on tertiles: low-volume (1–103 cases/year, n = 9712), intermediate-volume (104–195, n = 9664), and high-volume (196–679, n = 9700) groups. Table 1 presents the baseline patient characteristics of the volume groups. Significant differences were observed among the three groups in terms of patient demographic characteristics, hospital characteristics, comorbidities, and medications.

**Table 1:** Baseline patient characteristics according to hospital palliative care team intervention volume

Hospital palliative care team intervention volume^a^ (cases/year)	Low-volume group (1**–**103)		Intermediate-volume group (104**–**195)		High-volume group (196**–**679)		P-value^†^
N (%)	9712	(33.4)	9664	(33.2)	9700	(33.4)	
Demographic characteristics							
Admission year							
2015**–**2016	2775	(28.6)	2218	(23.0)	2128	(21.9)	< 0.001
2017**–**2018	3074	(31.7)	2622	(27.1)	3676	(37.9)	
2019**–**2020	3863	(39.8)	4824	(49.9)	3896	(40.2)	
Sex							
Male	4795	(49.4)	4413	(45.7)	4271	(44.0)	< 0.001
Female	4917	(50.6)	5251	(54.3)	5429	(56.0)	
Age, in years							
20**–**39	577	(5.9)	671	(6.9)	718	(7.4)	< 0.001
40**–**49	1155	(11.9)	1216	(12.6)	1341	(13.8)	
50**–**59	1785	(18.4)	1983	(20.5)	2023	(20.9)	
60**–**69	3055	(31.5)	2790	(28.9)	2895	(29.8)	
70**–**79	2564	(26.4)	2399	(24.8)	2265	(23.4)	
≥ 80	576	(5.9)	605	(6.3)	458	(4.7)	
Body mass index, in kg/m^2^							
≤ 18.4	2129	(21.9)	2234	(23.1)	2302	(23.7)	< 0.001
18.5**–**24.9	6061	(62.4)	5982	(61.9)	5923	(61.1)	
≥ 25.0	1452	(15.0)	1380	(14.3)	1446	(14.9)	
Unknown	70	(0.7)	68	(0.7)	29	(0.3)	
Smoking status							
Non-smoker	4804	(49.5)	4925	(51.0)	4887	(50.4)	0.038
Current/former smoker	3916	(40.3)	3747	(38.8)	3746	(38.6)	
Unknown	992	(10.2)	992	(10.3)	1067	(11.0)	
Barthel index							
100	7279	(74.9)	7454	(77.1)	7985	(82.3)	< 0.001
< 100	2249	(23.2)	2001	(20.7)	1627	(16.8)	
Unknown	184	(1.9)	209	(2.2)	88	(0.9)	
Hospital characteristics							
Type of hospital							
Community hospital	6482	(66.7)	4724	(48.9)	6004	(61.9)	< 0.001
Academic hospital	3230	(33.3)	4940	(51.1)	3696	(38.1)	
Number of hospital beds							
Small (45–653)	5346	(55.0)	3370	(34.9)	3830	(39.5)	< 0.001
Large (654–1475)	3096	(31.9)	4987	(51.6)	4450	(45.9)	
Unknown	1270	(13.1)	1307	(13.5)	1420	(14.6)	
Chemotherapy volume, in cases/year‡							
Low (16–1328)	5320	(54.8)	2307	(23.9)	2079	(21.4)	< 0.001
Intermediate (1329–2066)	2992	(30.8)	4130	(42.7)	2603	(26.8)	
High (2067–6242)	1400	(14.4)	3227	(33.4)	5018	(51.7)	
Comorbidities							
Charlson comorbidity index							
0**–**2	4522	(46.6)	4840	(50.1)	5173	(53.3)	< 0.001
≥ 3	5118	(52.7)	4752	(49.2)	4434	(45.7)	
Unknown	72	(0.7)	72	(0.7)	93	(1.0)	
Dementia	409	(4.2)	264	(2.7)	237	(2.4)	< 0.001
Brain tumor including metastasis	484	(5.0)	363	(3.8)	448	(4.6)	< 0.001
Cancer							
Lower gastrointestinal cancer	1026	(10.6)	797	(8.2)	706	(7.3)	< 0.001
Upper gastrointestinal cancer	935	(9.6)	833	(8.6)	867	(8.9)	
Lung cancer	2065	(21.3)	1511	(15.6)	1664	(17.2)	
Leukemia and lymphoma	869	(8.9)	1073	(11.1)	1575	(16.2)	
Other cancers	4817	(49.6)	5450	(56.4)	4888	(50.4)	
Medications							
Antibiotics	1291	(13.3)	1625	(16.8)	1645	(17.0)	< 0.001
Opioids	5486	(56.5)	5360	(55.5)	4000	(41.2)	< 0.001
Gabapentinoids	1787	(18.4)	1722	(17.8)	1452	(15.0)	< 0.001
Hypnotics	2848	(29.3)	3136	(32.5)	3031	(31.2)	< 0.001

^a^Annual number of new palliative care team interventions in hospitals

^†^Chi-square test

^‡^Annual number of inpatient chemotherapies in a hospital

Table 2 presents the results of the multivariate logistic regression analysis. A higher hospital PCT intervention volume was significantly associated with lower odds ratios of 30-day delirium (odds ratios: intermediate volume group, 0.79 [95% CI, 0.69–0.91]; high volume group, 0.80 [0.69–0.93]), 30-day mortality (intermediate volume group, 0.73 [0.60–0.90]; high volume group, 0.59 [0.46–0.75]), and decline in ADL (intermediate volume group, 0.77 [0.70–0.84]; high volume group, 0.52 [0.47–0.58]), considering the low-volume group as the reference. The variance inflation factors for all independent variables were < 4.0.

**Table 2:** Logistic regression results: odds ratios of each outcome for hospital palliative care team intervention volume

Hospital palliative care team intervention volume^a^	Low-volume group (1–103 cases/year) N = 9712	Intermediate-volume group (104–195 cases/year) N = 9664	High-volume group (196–679 cases/year) N = 9700
30-Day delirium	N = 548 (5.6%) OR = 1.00 (Ref)	N = 494 (5.1%) OR = 0.79 (0.69–0.91) *P* = 0.001	N = 453 (4.7%) OR = 0.80 (0.69–0.93) *P* = 0.003
30-Day mortality	N = 259 (2.7%) OR = 1.00 (Ref)	N = 180 (1.9%) OR = 0.73 (0.60–0.90) *P* = 0.003	N = 123 (1.3%) OR = 0.59 (0.46–0.75) *P* < 0.001
Decline in ADL	N = 1277 (13.1%) OR = 1.00 (Ref)	N = 1048 (10.8%) OR = 0.77 (0.70–0.84) *P* < 0.001	N = 701 (7.2%) OR = 0.52 (0.47–0.58) *P* < 0.001

Odds ratios are presented with their 95% confidence intervals and *P* values

*ADL* activities of daily living, *OR* odds ratio

^a^Annual number of new palliative care team interventions in hospitals

**Fig. 2:**
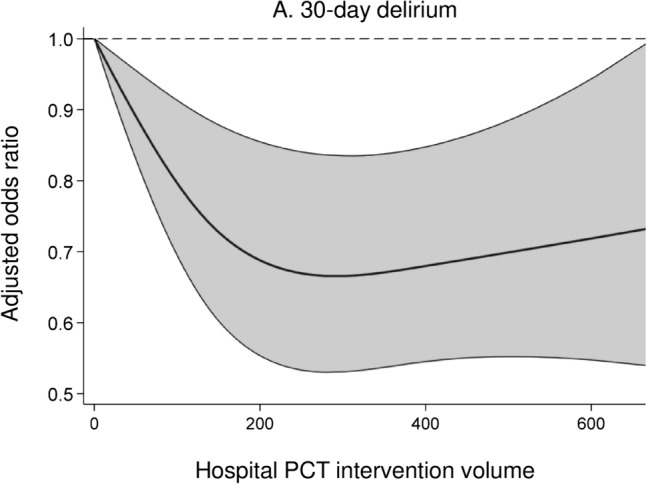
Results of the restricted cubic spline regression analysis. Association between continuous hospital palliative care team (PCT) intervention volume and the incidence of 30-day delirium (**A**), 30-day morality (**B**), and decline in activities of daily living (ADL) (**C**). The solid line represents the odds ratios, and the shaded area represents the 95% confidence intervals

Figure 2 presents the results of the restricted cubic spline regression analysis. Continuous hospital PCT intervention volume revealed significant inverse associations with the incidence of 30-day delirium (Fig. 2A), 30-day mortality (Fig. 2B), and decline in ADL (Fig. 2C). The odds ratios of all independent variables for each outcome are presented in eFigures 1–3.

## Discussion

The present study revealed a significant inverse association between hospital PCT intervention volume and the incidence of 30-day delirium, 30-day mortality, and a decline in ADL among hospitalized patients with cancer receiving chemotherapy.

Several randomized controlled trials have reported the benefits of palliative care in patients with advanced cancer [5–8]. These studies compared the differences between palliative care and usual care; however, the PCT skill was not considered. The present study revealed the association between higher hospital PCT intervention volume and better patient outcomes, suggesting that skilled PCT may improve patient outcomes. Medications administered to patients with advanced cancer occasionally cause life-threatening complications, including addiction, oversedation, and respiratory depression. The use of opioids, benzodiazepines, and antipsychotics was reported to result in fatal and severe harm outcomes in a review of all medication incidents reported to the National Reporting and Learning System in England [33]. Nausea, constipation, and delirium are some of the adverse effects of opioids that may lead to malnutrition and prolonged hospital stay, resulting in poor patient outcomes [34, 35]. Opioid overdose may cause oversedation and respiratory depression, resulting in cardiopulmonary arrest owing to hypoxia and hypercapnia [9]. Antipsychotic agents are often administered for the management of delirium; however, they can cause oversedation and oropharyngeal dysphagia, resulting in aspiration pneumonia and death [10, 11]. A recent randomized controlled trial reported that the use of antipsychotics for palliative care did not improve the incidence of delirium, increased extrapyramidal symptoms, and shortened patient survival compared with placebo. [36] Although benzodiazepines are also often used to manage uncontrolled agitated delirium, Hui et al. recommended that clinicians should prescribe appropriate medication at the appropriate dose with attention to the considerable risk of oversedation [37]. Inappropriate use of opioids, antipsychotics, and benzodiazepines can cause delirium, oversedation, aspiration pneumonia, respiratory depression, and death. Therefore, appropriate management of these medications by a skilled PCT may lead to improved patient outcomes.

The required volume of hospital PCT interventions for favorable patient outcomes remains unknown owing to the lack of prior studies. The graphs of odds ratios for delirium, mortality, and decline in ADL obtained in the restricted cubic spline regression revealed a rapid downward trend of up to approximately 200 cases/year (Fig. 2). However, hospital PCT intervention volume was the total number of patients with cancer on chemotherapy who received PCT intervention, and did not include patients without cancer or postoperative patients with cancer who received PCT intervention. Therefore, it was difficult to determine the sufficient hospital PCT intervention volume required for favorable patient outcomes based on this study.

There might be an opinion that the favorable outcomes for patients are due to the large size of the hospital. Indeed, Table 1 shows that the groups with higher hospital PCT intervention volume tend to have a larger number of hospital beds and a higher chemotherapy volume. However, the influence of these potential confounders has been adjusted by including these covariates in the logistic regression model. Therefore, the favorable outcomes for patients were considered to be associated with the higher hospital PCT intervention volume, not with the size of the hospital.

The relationship between surgical skills and patient outcomes has been well investigated. The hospital surgical volume was often used as an alternative quality indicator of surgical skill in these studies. For instance, volume-outcome associations have been observed in cardiac, spinal, pancreatic, and urological surgeries [13, 38–40]. However, to the best of our knowledge, no similar studies have been conducted in the field of palliative care. This study used methods similar to those used in previous studies in the field of surgery to assess the volume-outcome association of the hospital PCT intervention volume. Further studies must be conducted to validate the association between hospital PCT intervention volumes and PCT levels.

This study has some limitations. First, the validity of the association between PCT skills and hospital PCT intervention volume is unclear. Second, the validity of the delirium identification algorithm based on the use of haloperidol and risperidone is lower than that of a diagnosis based on a scoring system, such as the Confusion Assessment Method. However, a previous study reported that the delirium-identification algorithm based on the use of antipsychotics had relatively high sensitivity (64%), specificity (97%), and positive predictive value (83%) for identifying hyperactive or mixed delirium in an inpatient administrative database [16]. Third, the recorded diagnoses in administrative databases are less validated than those in planned prospective cohort studies. Fourth, the outcomes set in this study were not exactly ideal outcomes for measuring PCT skills. Ideal outcomes for measuring PCT skills would include patient satisfaction, patient QOL, and the burden on medical staff. Unfortunately, these data were not included in the database. Meanwhile, development of delirium and decline in ADL can reduce patient QOL and impose a significant burden on medical staff. Therefore, delirium and ADL were considered to serve as surrogates for ideal outcomes. Lastly, potential confounders, such as race, education level, economic status, and cancer stage, were not included in the statistical model of this study, and therefore the influences of these factors were unadjusted.

The present study revealed associations between higher hospital PCT intervention volume and lower odds ratios for 30-day delirium, 30-day mortality, and decline in ADL among hospitalized patients with cancer receiving chemotherapy. The odds ratios showed rapid downward trends of up to approximately 200 cases/year in the hospital PCT intervention volume. Our findings suggest that intervention performed by skilled PCT may lead to improved patient outcomes. However, even if this is true, aggregating patients in high volume hospitals may not always be the best solution. It would be rather important to appropriately allocate well-trained palliative care specialists in each hospital.

## Supplementary Information

Below is the link to the electronic supplementary material.

Supplementary file1 (TIF 711 KB)

Supplementary file2 (TIF 709 KB)

Supplementary file3 (TIF 711 KB)

## Data Availability

Data are not available due to contractual reasons.
