# Supplementary figures and images for: Association between hospital palliative care team intervention volume and patient outcomes
Source: Int J Clin Oncol. 2024 Jun 24;29(10):1602–9. doi: 10.1007/s10147-024-02574-4 (PMC11420267; doi:10.1007/s10147-024-02574-4)

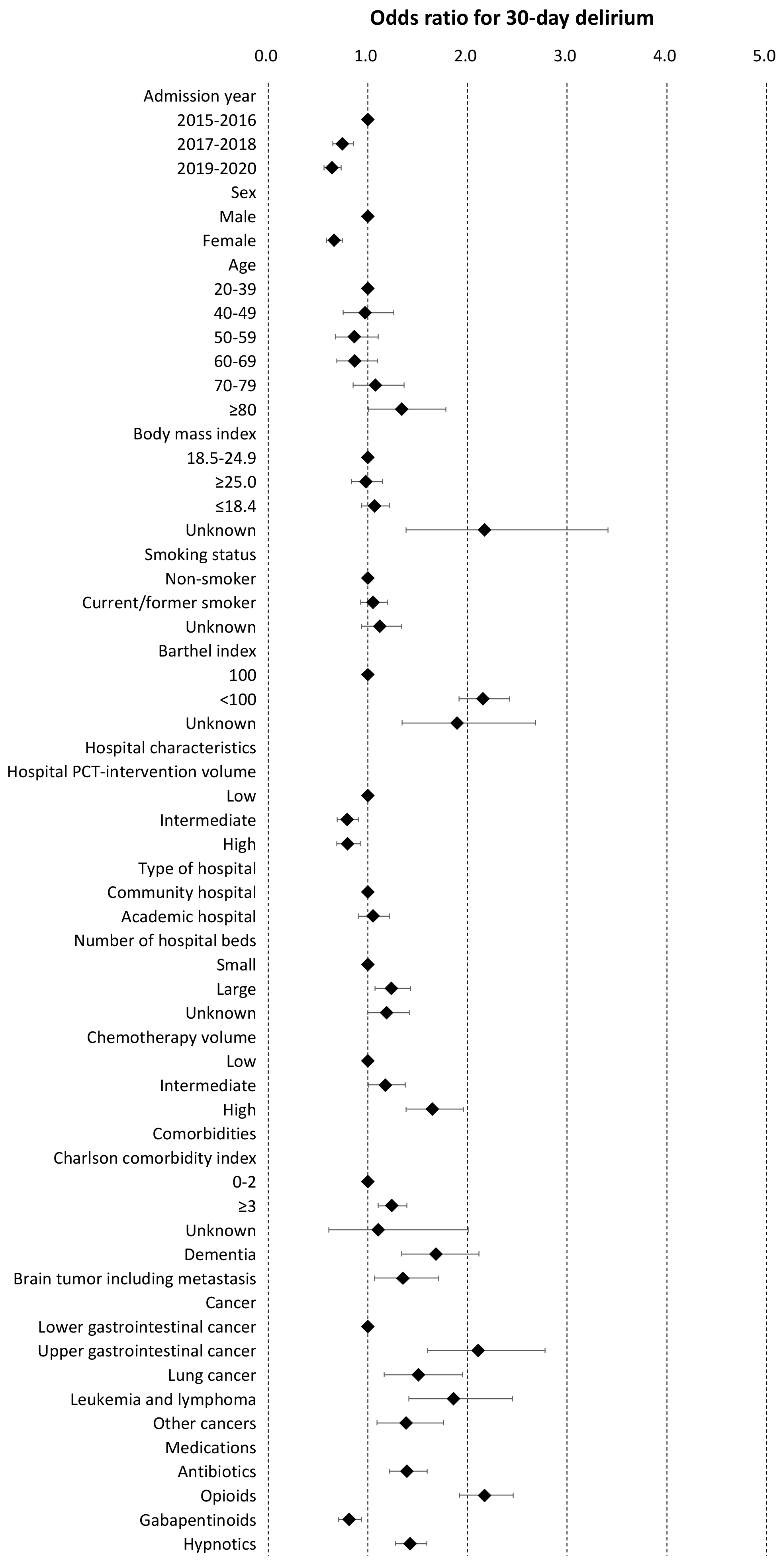

Supplement: Supplementary file 1 — Supplementary file1 (TIF 711 KB) [file 10147_2024_2574_MOESM1_ESM.tif]

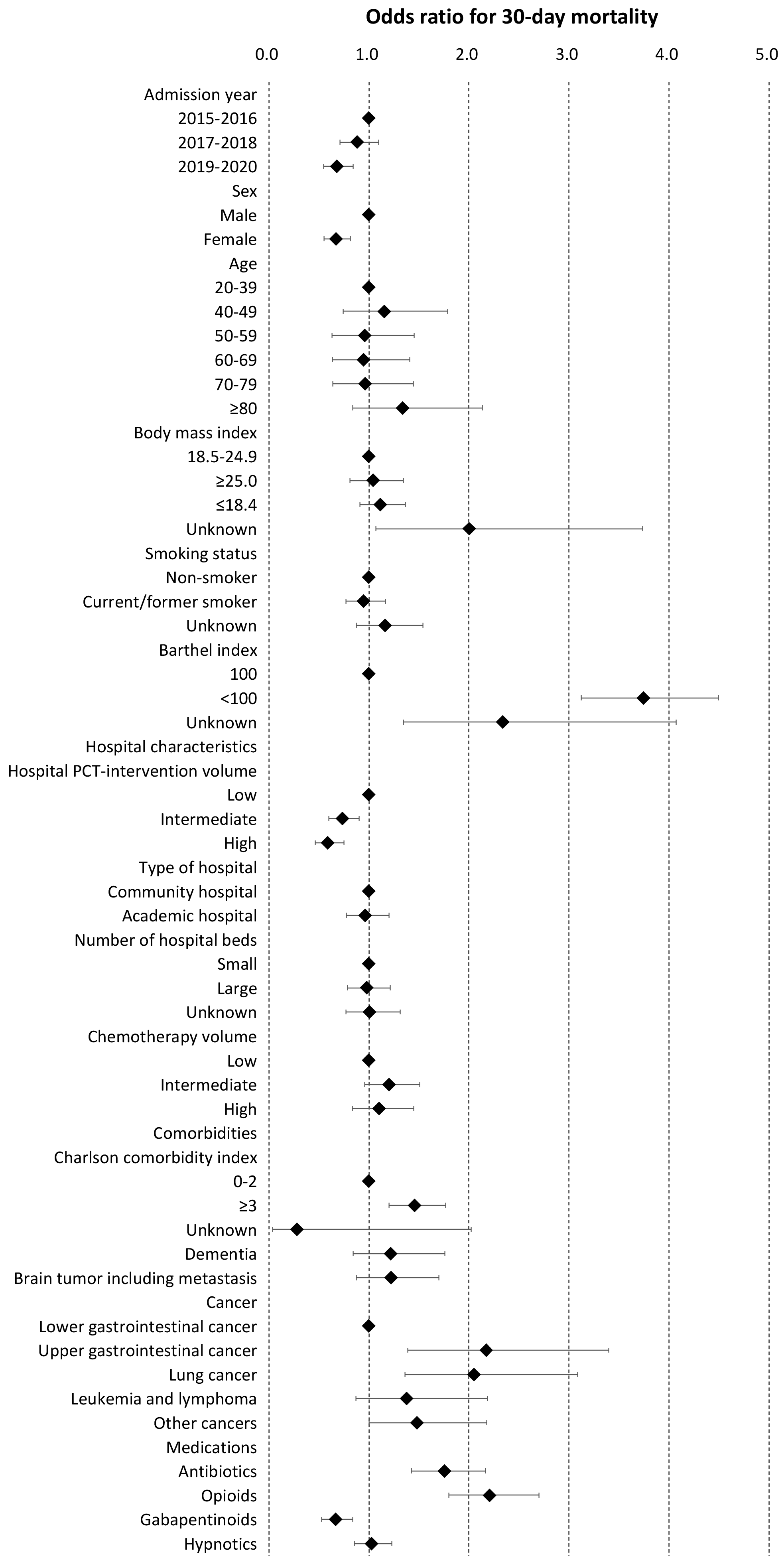

Supplement: Supplementary file 2 — Supplementary file2 (TIF 709 KB) [file 10147_2024_2574_MOESM2_ESM.tif]

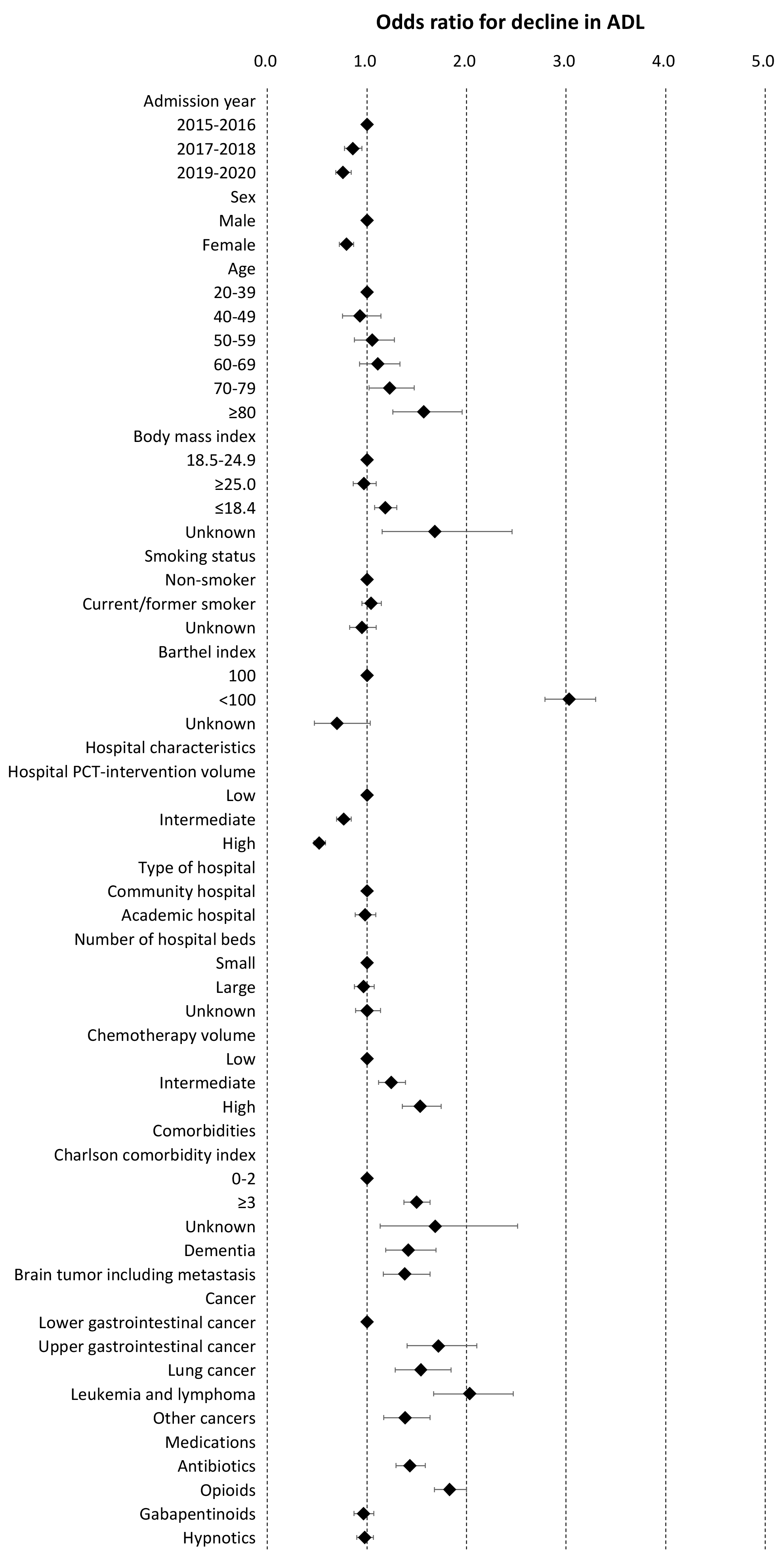

Supplement: Supplementary file 3 — Supplementary file3 (TIF 711 KB) [file 10147_2024_2574_MOESM3_ESM.tif]
